# The Operation for the Cure of Double Hare Lip, by a New and Improved Method

**Published:** 1868-01

**Authors:** A. Hammer


### SELECTED ARTICLES.

### ARTICLE VI.

### *The Operation for the Cure of Double Hare Lip, by a New and Improved Method.*

By A. Hammer, M.D.

During a quarter of a century I have had frequent occasion to operate for hare-lip, in all its various forms, single, double and complicated ; and I freely confess that for twenty years I was never satisfied with the results obtained, though mine were, on the average, not worse than those of other Surgeons. I was frequently amused by looking at plates, where cases of hare-lip were pictured, before and after operation, showing beautiful and perfect results, whereas the comparison between the copy and the original would not have given a very flattering impression as to the ability or truthfulness of the artist.

The unsatisfactory results obtained in my own former practice, and present practice of other Surgeons, did not, and do not depend so much on the want of individual skill, as upon the intrinsic difficulties inherent to the nature of the lesion itself, and the deficiencies of the means employed to correct the deformity. The main points to which the frequent failures in double hare-lip with fissure of palate must be attributed, are : The rarity of union by first intention in the soft parts, or union of one portion with non-union or connection by ligamentous mass of the remainder ; the infrequency of firm union of the intermaxillary bones with lateral alveolar arches, and the resulting unevenness by lack of proper adaptation with regard to the convexity of the entire superior alveolar arch ; the frequent mutilation of the nares, either by closing them up, or leaving them widely separated, the flat nose in the superlative.

Nearly all the difficulties with which the surgeon has to contend, can be overcome by following the method of operating which I have adopted.

The operative procedure consists of two steps : First, to bring the maldirected, intermaxillary bones into proper position and to make them fit exactly the opening left in the middle of the alveolar arch. This I accomplish by excising a triangular piece of the septum of the nose, of such an angle as to correspond to the angle made by the projecting inter-maxillary bones with the arch. After it has gently been moved downwards and backwards, the surgeon can judge how much or how little is to be cut off on one side or both, that the gap may be exactly closed. I give preference to this method of changing direction over all others.

Second : To separate, as may be required, the middle lobe from the intermaxillary bones, then to freshen its edges as well as the margins of the lateral parts of the lip, resorting if necessary to auxillary incisions in various directions according to the peculiarities of the shortening in the soft parts, accompanied by free and extensive incisions over the underlying bone so as to allow of great mobility of the lip. This being done, and the hemorrhage arrested, I apply a sustaining suture, which is in fact a quill-wire-suture, at a proper distance from the edges to be united. Two pieces of common, smooth lead pencil, from one and a half inch to one and three-fourths of an inch in length, and a strong needle armed with a double wire of a size larger than is ordinarily employed in the usual wire suture, are all that will be required.

The needle is passed through the entire thickness of the upper lip on a transverse line, striking the point of union between the septum and intermaxillary bones. The needle is made to transfix the integument from without inwards on one side, at a point half an inch posterior or outwards from the nostril, and through a corresponding point, but from within outwards, on the opposite side, and now the two pieces of pencil, one on either side of the face externally, are fastened by the double wire. Another similar suture is applied in the same manner and attached to the same pieces of pencil, about half an inch below the first, more near or remote according to the length of the intermaxillary bones, over which, that is to say in front of which, both wires must pass. By this means we accomplish a complete relaxation of the soft parts, all tension of the muscles being overcome the corresponding portions of the cut edges can now be readily approximated, to do which I employ the common wire suture—the wire being very small,—finding it less irritating than silk. Thus the operation is completed, no dressing being required except the occasional application of a little glycerine by means of a camils hair pencil, upon the united wounds. The wire suture should be removed at the end of three days, union by first intention having then taken place, while the sustaining suture may be allowed to remain to the sixth, seventh, eight or ninth day. The wires of the latter in course of time cut somewhat the soft parts, producing four small, transverse, slightly supurating wounds, which, however, heal without leaving any marked scar behind.

The advantages of the above plan of procedure are so obvious that I need scarcely refer to them, but in brief they are the following:

First, The intermaxillary bones are kept in close contact with the parts with which it is desirable they should unite, by the wires of the sustaining suture.

Second, All strain on the lips being removed, the soft parts must unite by first intention, it cannot be otherwise provided all chemical or mechanical irritants are wiped from the wounds, which can so readily be done by a hair pencil.

Third, The degree of relaxation necessary to properly control and modify the future shape of the nares is entirely at the command of the surgeon.

Fourth, The absence of all dressing which would interfere with free respiration and thereby endanger life.

Fifth, The operation is completed at one session, and comparatively speaking, very brief space of time is required for complete and permanent union.

Sixth, The surgeon is relieved from an immense deal of trouble and constant attention, which is so necessary when other operative plans of treatment are adopted.

Seventh, The results are admirable, thereby not saying too much.

This method is not altogether new, as it has been resorted to, but only partially and for a different object, by Prof. Bruns, of Tubingen. Many years ago he applied a sort of quill suture, passing out one such beneath the nostrils through the septum narium to prevent too great narrowing of the nares, and in one instance he again applied a single quill suture near the free margin of the lip, in an unmangeable child, lest the lower suture when removed might be followed by rupture of the united wound. His fear in this last instance was certainly to some extent groundless, for in five cases out of six the rupture occurs, not near the free margin, but in the neighborhood of the nares.

The actions mainly of two muscles, viz: the levator labii superioris alæque nasi and the levator labii superioris proprius, has to be overcome. The zygomatici and the levator anguli oris are little to be feared, as any one can convince himself by applying his index fingers to the two sides of his lips, imitating my sustaining suture.

Though the meritorious and highly distinguished Prof.

Bruns did not apply the quill suture either in the same manner or for the same purpose, yet I thought it my duty to show that I was acquainted with the fact though irrelevant.

I earnestly desire the profession to give my modus operandi a trial, being assured it will meet with their approval. Of myself I can, without boasting, affirm that I am not now fearful of any form of complicated hare-lip, no matter how extreme the case may be, and that I now with pleasure and satisfaction perform an operation which formerly caused me more disappointment than any other one. —*Humboldt Medical Archives.*

